# Enhancement of the leptomeninges in a young man

**DOI:** 10.1111/bpa.70145

**Published:** 2026-09-16

**Authors:** Jayden Ray, Mohammad Alberawi, Andrew Bauer, Kar‐Ming Fung, Erika Santos Horta, Lorraina J. Robinson

**Affiliations:** ^1^ Department of Pathology University of Oklahoma Oklahoma City Oklahoma USA; ^2^ Department of Neuroradiology University of Oklahoma Oklahoma City Oklahoma USA; ^3^ Department of Neurosurgery University of Oklahoma Oklahoma City Oklahoma USA; ^4^ Department of Neuro Oncology University of Oklahoma Oklahoma City Oklahoma USA

**Keywords:** borderline tumor, melanocytic neoplasm, neuropathology, *NRAS* and *EIF1AX*‐mutant, primary diffuse leptomeningeal melanocytosis and melanomatosis

1

BOX 1 **Virtual glass slide.**

Access at https://isn‐slidearchive.org/?col=ISN&fol=Archive&file=20260528.svs.

## 2 CLINICAL HISTORY AND IMAGING

**FIGURE 1:**
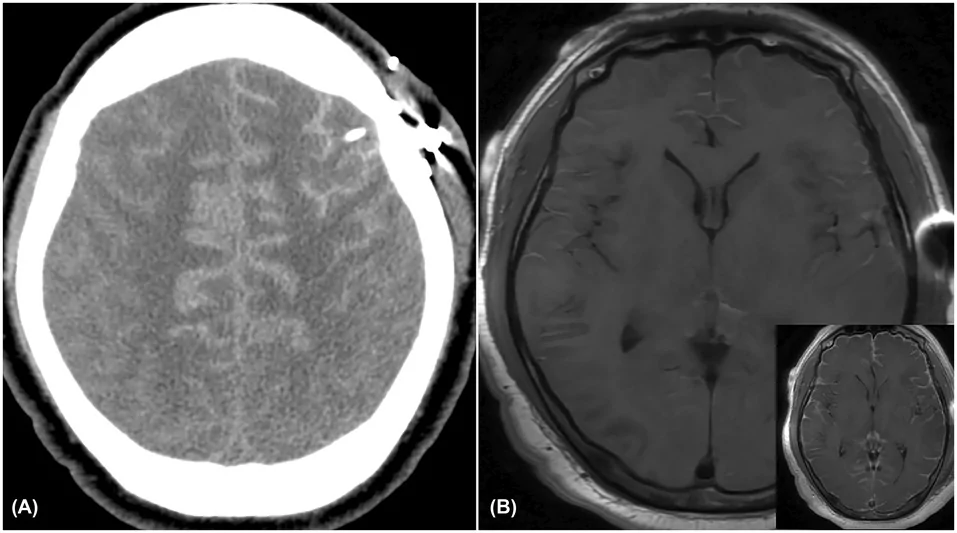
Imaging features. (A) Non‐contrast CT reveals increased density of the sulci primarily in the frontal lobes. (B) MRI axial T1 pre‐contrast shows increased intrinsic T1 signal along the sulci. Inset (lower right): axial T1‐weighted post‐contrast MRI reveals prominent, superimposed diffuse leptomeningeal enhancement.

This case involves a 22‐year‐old male with a past medical history significant for hypertension, who was transferred from an outside hospital on investigation of multiple prior nontraumatic subarachnoid hemorrhages and status epilepticus. Initial non‐contrast computed tomography (CT) revealed increased sulcal density primarily in the bilateral frontal lobes mimicking a subarachnoid hemorrhage (Figure 1A). Subsequent brain magnetic resonance imaging (MRI) revealed T1 intrinsic hyperintensity of the bilateral cerebral sulci with diffuse superimposed leptomeningeal enhancement along the sulci, surface of the brainstem, cranial nerves, and ventricular margins (Figure 1B). A left frontal craniotomy was pursued for biopsy and definitive diagnosis. Intraoperatively, after the dura was opened, a dark, tar‐like appearance involving the surface of the brain was visualized. Lesional tissue was obtained and sent for pathological analysis.

## 3 FINDINGS

**FIGURE 2:**
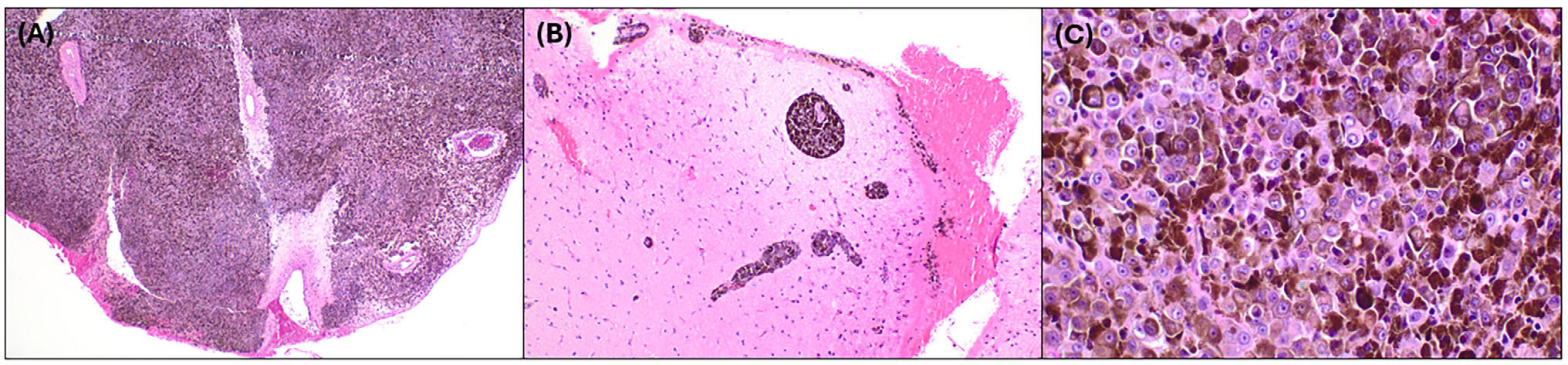
Histological features. (A) Tumor sections show a hypercellular, pigmented epithelioid neoplasm (4×). (B) Involvement of the tumor within the Virchow‐Robin spaces without definitive invasion (10×). (C) The tumor cells displayed prominent nucleoli, eosinophilic cytoplasm, moderate to severe nuclear atypia, and contained dense, granular, brown pigment (40×).

Histological analysis of the H&E‐stained sections demonstrated a diffuse leptomeningeal proliferation of highly pigmented epithelioid melanocytic cells with moderate to severe nuclear atypia and infrequent mitotic figures (Figure 2A,C, Box 1). Necrosis was absent. Extension into the Virchow‐Robin spaces without definitive parenchymal invasion was noted (Figure 2B). Immunohistochemistry staining revealed the tumor cells to be positive for Melan‐A with preserved nuclear BAP1 expression. Clinically, the patient did not have a history of cutaneous melanoma or neurocutaneous syndrome.

Next‐generation sequencing revealed an activating *NRAS* p.Gln61His (Q61H) mutation and a hotspot *EIF1AX* p.Gly8Arg mutation in exon 1. Whole genome methylation profiling was performed with a composite methylation profile listed as “No Match.” The corresponding copy number plot revealed a shallow gain of chromosome 8 and shallow deletions of 9q and 11q (focal), while 9p21 (*CDKN2A/B*) and 3p appeared intact.

Given the concerning imaging findings of a diffuse leptomeningeal process, and by applying the Central Nervous System‐World Health Organization (CNS‐WHO) framework, diffuse primary leptomeningeal melanocytic neoplasms (PLMNs) are classified as melanocytosis (benign) or melanomatosis (malignant) based on atypia, mitotic activity, invasion, and necrosis. In this case, the lesion displayed nuclear atypia and exceeded the cytologic blandness expected for classic meningeal melanocytosis. However, the low mitotic activity and lack of necrosis made the lesion fall short of overt melanomatosis. Histologically, this placed the lesion within a borderline zone between melanocytosis and melanomatosis.

The combination of clinical history, radiological findings, tumor morphology, and molecular findings was determined to be more consistent with a PLMN than with a conventional metastatic cutaneous melanoma. Overall, the findings support a borderline diffuse leptomeningeal melanocytic neoplasm, *NRAS*‐ and *EIF1AX*‐mutant, with potential for aggressive behavior.

## 4 FINAL DIAGNOSIS

Primary diffuse leptomeningeal melanocytic neoplasm, *NRAS* and *EIF1AX*‐mutant, borderline between melanocytosis and melanomatosis.

## 5 DISCUSSION

Primary melanocytic tumors involving the CNS represent a spectrum of very rare tumors that can be classified as either malignant or benign. These tumors can present clinically as either a focally well‐circumscribed lesion or a diffuse lesion with widespread involvement along the leptomeninges [1]. As defined by the most recent 2021 CNS‐WHO tumor classification system, meningeal melanocytosis is a diffuse or multifocal meningeal proliferation of cytologically bland melanocytic cells, while meningeal melanomatosis is a diffuse or multifocal meningeal proliferation of melanotic cells that often show invasion into the CNS parenchyma. Both entities arise from native leptomeningeal melanocytes derived from neural crest cells.

From an epidemiological standpoint, diffuse meningeal melanocytic neoplasms are very rare, making population‐based studies challenging to assess and quantify. These tumors have been reported to occur in both adults and children. Melanocytosis primarily affects children in the setting of neurocutaneous melanosis (NCM) [1]. Melanomatosis often exhibits a bimodal age distribution and may manifest in children, with or without NCM, and in adults predominantly in the 4th decade of life [1].

It has been proposed, in a study by Kinsler et al., that the pathogenesis involving meningeal melanomatosis and melanocytosis is predominately associated with postzygotic mutations in the *NRAS* gene at codon 61, which results in an initial genetic insult via proto‐oncogene activation where the *NRAS* gene acts as a primary driver in a multi‐step oncogenic process [2]. The *NRAS* gene is an important member of the RAS GTPase family that primarily regulates cellular proliferation, differentiation, and survival involving the RAF/MEK/ERK and PI3K/mTOR pathways [1].

The present case also showed an *EIF1AX* p.Gly8Arg mutation in exon 1. According to a study by Küsters‐Vandevelde et al., detection of missense mutations involving *EIF1AX* (exon 1 and 2 and flanking intronic regions) was found to occur at a relatively high frequency (~21%) in PLMNs. These mutations were also observed generally co‐occurring with *GNAQ*, *GNA11*, and *NRAS* mutations, suggesting that the *EIF1AX* mutations arise later in the tumorigenesis pathway [3].

This interesting case demonstrates a rare tumor with unique histological features where significant overlap was observed between melanocytosis and melanomatosis. The tumor could not be placed in either category, making it a rare borderline diffuse leptomeningeal melanocytic neoplasm, *NRAS*‐ and *EIF1AX*‐mutant, with potential for aggressive behavior.

## AUTHOR CONTRIBUTIONS

*Clinical data collection*: JR, MA, AB, KF, ESH, and LJR. *Figures and manuscript text preparation*: JR, MA, AB, KF, ESH, and LJR. *Study supervision*: LJR. All authors made substantial contributions to the conception or to the acquisition, analysis, or interpretation of data. All authors reviewed and approved the final manuscript.

## CONFLICT OF INTEREST STATEMENT

The authors declare no conflicts of interest.

## Data Availability

Data sharing is not applicable to this article as no new data were created or analyzed in this study.
